# Perceptions about the acceptability and prevalence of HIV testing and factors influencing them in different communities in South Africa

**DOI:** 10.1080/17290376.2014.937355

**Published:** 2014-07-25

**Authors:** Yoliswa Ntsepe, Leickness C. Simbayi, Olive Shisana, Thomas Rehle, Musawenkosi Mabaso, Nolusindiso Ncitakalo, Alicia Davids, Yogandra Dhee Naidoo

**Affiliations:** ^a^ MA, PhD Intern at the HIV/AIDS, STIs and TB Unit, Human Sciences Research Council, Cape Town, South Africa; ^b^ DPhil, is Executive Director at the HIV/AIDS, STIs and TB Unit, Human Sciences Research Council, Cape Town, South Africa; ^c^ Sc.D, Chief Executive Officer of the Human Sciences Research Council, Cape Town, South Africa; ^d^ MD, MSc & PhD, is Senior Advisor at the HIV/AIDS, STIs and TB Unit, Human Sciences Research Council, Cape Town, South Africa; ^e^ PhD, is Chief Research Specialist at the HIV/AIDS, STIs and TB Unit, Human Sciences Research Council, Durban, South Africa; ^f^ MA, is PhD Intern at the HIV/AIDS, STIs and TB Unit, Human Sciences Research Council, Cape Town, South Africa; ^g^ MA, is Research Specialist at the HIV/AIDS, STIs and TB Unit, Human Sciences Research Council, Cape Town, South Africa; ^h^ MA, is PhD Intern at the HIV/AIDS, STIs and TB Unit, Human Sciences Research Council, Durban, South Africa

**Keywords:** HIV Counselling and Testing, perceptions, stigma, discrimination and confidentiality, South Africa, Le conseil et le depistage du VIH, Perceptions, stigmate, discrimination, confidentialite, Afrique du Sud

## Abstract

HIV counselling and testing (HCT) is considered important because it is an entry point to a comprehensive continuum of care for HIV/AIDS. The South African Department of Health launched an HCT campaign in April 2010, and this reached 13,269,746 people by June 2011, of which 16% tested HIV positive and 400,000 of those were initiated into antiretroviral treatment. The overall objective of this project was to gain insight into the general perceptions about HIV testing in the different South African communities. Factors influencing testing in these communities were also explored. Discussions with twelve focus groups (FG) of 8–12 participants each were conducted with male and female participants recruited from both urban formal and informal communities in Cape Town and Durban. Participants included four racial groups represented by different age groups as follows: adolescents (12–17 years), youth (18–24 years) and adults (25 years and older). Data were analyzed using thematic coding. Among the key themes that emerged from the findings were the inaccurate perception of risk, fear of testing HIV positive, stigma and discrimination. Participants from both African and Indian FGs reported being less likely to do self-initiated HIV testing and counselling, while those from the FG consisting of young whites were more likely to learn about their HIV status through blood donations and campus HIV testing campaigns. Most FGs said they were likely to test if they understood the testing process better and also if the results are kept confidential. The present findings reiterate the importance of spreading positive messages and ensuring confidentiality for HIV testing in a society where there is still some stigma associated with people living with HIV/AIDS. This can partly be accomplished by the continuation of the national HCT campaign, which has been a considerable success in the fight against HIV/AIDS in South Africa during the past two years.

## Résumé

Le conseil et le dépistage (CDV) du VIH sont considérés importants pour les programmes de soins du VIH/SIDA. Le ministère sud-africain de la Santé a lancé une campagne de CDV en avril 2010, et a atteint 13 269 746 personnes en juin 2011, dont 16% ont été identifiés séropositifs, et dont 400,000 ont commencé le traitement antirétroviral. L'objectif global de ce projet était de mieux comprendre les perceptions générales sur le dépistage du VIH dans les différentes communautés sud-africaines. Les facteurs influençant la participation au dépistage du VIH ont été aussi étudiés. Douze groupes de discussion (GDD), consistant de huit à douze hommes et femmes, ont été formés. Les participants étaient recrutés dans les localités urbaines formelles et informelles à Cape-Town et à Durban. Les groupes étaient repartie en quatre sur base d'ethnicité, représentant différents groupes d’âge: les adolescents (12–17 ans), les jeunes (18–24 ans) et les adultes (25 ans et plus). Les données ont été analysées en utilisant un codage thématique. La perception erronée du risque, la peur du dépistage du VIH, la stigmatisation et la discrimination étaient parmi les principaux thèmes qui ont émergé. Les participants africains et indiens ont déclaré d’être moins disposés à se faire tester pour le VIH à propre initiative, tandis que les jeunes blancs étaient disposés à connaître leur statut VIH grâce aux dons de sang et aux campagnes universitaires de dépistage du VIH. La plupart des participants des DDG ont dit qu'ils étaient disposés à se laisser tester s'ils avaient une meilleure compréhension du processus de dépistage, et s'ils étaient convaincus de sa confidentialité. Les résultats de cette recherche ont réitéré l'importance de la diffusion de messages positifs et de la confidentialité des résultats le dépistage du VIH dans une société où il y a encore un stigmate associé au VIH. Cela peut être accompli en partie par la poursuite de la campagne nationale de CDV, qui a été un succès considérable dans la lutte contre le VIH/SIDA en Afrique du Sud au cours des deux dernières années.

## Background

South Africa has the highest number of people living with HIV/AIDS (PLWHA) in the world with an estimated 6.4 million of its citizens reported as living with HIV/AIDS in 2012 (Shisana, Rehle, Simbayi, Zuma, Jooste, Zungu, *et al*. 2014). The HIV pandemic in South Africa was reported to have stabilized between 2000 and 2005 (Shisana, Rehle, Simbayi, Zuma, Jooste, Pillay, *et al*. 2005, 2009; Shisana & Simbayi 2002) and by 2008 the country experienced a significant decline in HIV incidence (Rehle, Hallet, Shisana, Pillay-van Wyk, Zuma, Carrera, *et al*. 2010). This was attributed to the reported significant increase in condom use and awareness of HIV status, especially among youth. The increase in condom use can be attributed to the extensive national social and behavioural change communication campaigns as well as the schools' life skills programmes. The increase in awareness of HIV status was achieved through a national HIV Counselling and Testing (HCT) campaign that aimed to counsel and test 15 million South Africans for HIV. This HCT campaign was conducted between April 2010 and June 2011 as part of the previous National Strategic Plan for HIV/AIDS and Sexually Transmitted Infections (STIs) for 2007–2011 (Department of Health 2007; Mayosi, Lawn, van Niekerk, Bradshaw, Abdool Karim, Coovadia for the Lancet South African team 2012; Motsoaledi 2012).

During this HCT campaign, a record 13,269,746 HIV tests were conducted and 2,155,312 people (16%) were tested positive, of whom 48% had CD4 cell counts above 350 (Mbengashe, Nevhutalu, Chipimo, Chidarikire & Diseko 2012). During the HCT campaign over 400,000 patients were initiated on antiretroviral treatment (ART), of whom 57,000 were pregnant women (Mbengashe *et al*. 2012). According to Shisana *et al*. (2014), about half of the South African population had been tested for HIV by 2012. As a result of the recent national HCT campaign, South Africa now has one of the highest proportions of people who have tested for HIV and know their status in the world. Only Kenya and Botswana have achieved greater success, with 72.0% and 70.2% of their respective populations having tested for HIV (see National AIDS and STI Control Programme, Ministry of Health, Kenya 2013; Statistics Botswana 2013).

HCT is important because it is an entry point to a comprehensive continuum of care for HIV/AIDS. In particular, it plays a pivotal role in the population's response to HIV and is also a crucial entry strategy into HIV/AIDS services such as prevention of mother-to-child transmission (PMTCT), provision of ART, management of HIV-related illnesses and provision of psychosocial support (Chersich & Temmerman 2008; Menzies, Abang, Wayenze, Nuwaha, Mugisha, Coutinho, *et al*. 2009; SANAC 2010; Sherr, Loopman, Kakowa, Dube, Chawira, Nyamukapa, *et al.* 2007). Furthermore, De Cock, Mbori-Ngacha and Marun (2002) have established that an additional benefit of HCT is that it may potentially result in a decrease in HIV-related stigma, leading to a ‘normalization’ of the HIV epidemic.

HIV-related stigma is one of the most serious obstacles in the fight against HIV/AIDS the world over, including in South Africa. This is due to the fact that HIV infection, as with other STIs, is widely perceived as an outcome of sexual excess and low moral character (Leclerc-Madlala, Simbayi & Cloete 2009). At the time when those infected really need social support the most, PLWHA whose status is revealed are often subjected to victimization and discrimination. This happens in all social realms, in the home, in the workplace and in the broader community (Leclerc-Madlala *et al*. 2009). Consequently, there is a strong culture of silence and non-disclosure of positive status by PLWHA because of the fear of rejection and isolation from both close relatives and the community at large (Johnston 2001). The stigma is more severe for women than for men (Leclerc-Madlala *et al*. 2009; Petros, Airhihenbuwa, Simbayi, Ramlagan & Brown 2006).

The importance of HCT both as an entry point to a comprehensive continuum of care for HIV/AIDS and due to its potential to decrease HIV-related stigma motivates this paper's investigation of the acceptability and prevalence of HIV testing in South Africa. In this paper we also investigated the factors that influence HIV testing in different South African communities. This was done in order to ascertain whether HIV testing is now accepted as a norm in different South African communities as this would have some implications for the idea of providing feedback on HIV test results in a population-based survey that was planned for 2012 by the same research team.

## Methodology

### Participants recruitment

Twelve focus groups (FGs) were conducted with male and female participants 12 years of age and older drawn from four race groups (Africans, Coloureds, Indians and Whites) in two large cities of South Africa, namely, Cape Town and Durban. The information sheet about the study and the consent/assent forms were translated into different languages used in a particular community and distributed with the consent of the gatekeepers. Five pairs of FG facilitators, each consisting of one male and one female, were matched with the different communities depending on their language efficiency and racial group. This was done to encourage opening up during the FG discussions.

The snowball recruitment approach was used and venues were selected in such a way that none of the participants incurred transport costs. The gatekeepers of the various communities and schools were approached and introduced to the study. Participants who were aged 18 years and older were provided with an information sheet and were asked to sign a consent form agreeing to participate and also to be recorded during the FG discussion. The governing bodies of schools agreed for sending home to the parents and/or guardians of children between the ages of 12 and 17 years both a copy of the information sheet which explained in detail what the study objectives were and a consent form in order for the parents to consider whether or not they wished their children to participate in the study. The parents and/or guardian's permission was also sought for the FGs to be audio recorded to facilitate transcription. Those parents who agreed signed the consent forms after which their children too were also given an age-appropriate information sheet and assent form to sign before they participated in their respective FGs if they agreed to do so. The children also had to consent to be recorded during the FG discussion. There were no incentives for participation, but refreshments were served at the end of the FG discussions.

Each FG consisted of about 8–12 participants chosen from specified communities in urban formal and urban informal areas. All groups consisted of both male and female participants in three different age categories, namely, adolescents (12–17 years), youth (18–24 years) and adults (25 years and above). The snowball sampling approach used resulted in mostly university students comprising both Indian and White youth FGs, while the African and Coloured youth FG participants were mainly unemployed. A few of the adult participants were mostly working full time, while others worked in part-time employment.

There were no additional exclusion criteria; anyone who identified within the specified four race groups, was aged 12 years or older and resided in Durban or Cape Town within the specified communities where FGs were held was welcome to participate.

### Research method

The FG method is one of the most widely used qualitative data collection tools in social research (Creswell 2003; Patton 1987). Overall, a qualitative approach was chosen to enable the researchers to explore deep and rich information and to ensure that the participants' creativity of responses was not contained. This brings forth the primary meaning, showing how people made sense of concepts that evolve from their perceptions (Creswell 2003). A qualitative approach allows for the emergence of views that are specific to the sociocultural context of the participants. People derive meanings of situations that are typically forged in discussions or interactions with other persons (Creswell 2003).

### Instruments

A FG guide was used by all the researchers during data collection to ensure that the same questions were asked and similar types of information were obtained from the different race groups. The FG guide included questions that covered several issues pertaining to the acceptability of different HCT approaches and different procedures for providing HIV test result feedback to participants in a national survey. Three specific questions from the FG guide were analyzed in this paper in order to determine whether HIV testing was becoming a norm in South Africa. They include the following:

- What proportion of people in your community do you think have undergone HIV testing?
- (*If high*) Why do you think they have they done so?
- (*If low*) Why have they not done so? How could they be encouraged to do so?

### Procedure

The FGs were conducted in Afrikaans, English, Xhosa and Zulu depending on the city and language preference of the group. The FG facilitators were both racially and language matched with the participants in all the FGs. All discussions were recorded over a three-month period from August to October 2010. During the FGs, the co-facilitator took field notes, which were later used to validate transcripts for accuracy. Sessions were audiotaped and transcribed verbatim as well as translated into English where necessary.

### Analysis

Data were analyzed using thematic coding (Miles & Huberman 1994; Patton 1987). Immediately after conducting FGs in the field, each pair of researchers facilitating the FG wrote a report reflecting on the themes that emerged during the discussion. They then listened to the tape and looked at the notes that the other researcher took as supplementary to the recording. When the final transcripts were obtained, they were shared with other researchers for analysis and for comparing notes of emerging themes. This first level of analysis where researchers breakdown and examine the raw data and compare emerging themes is referred to as open coding (Kendall 1999; Strauss & Corbin 1990). Upon agreeing on the themes the research team met to discuss the consistent themes, followed by secondary coding which involved categorizing data derived from FG transcripts into meaningful themes. In practice, themes serve to identify, label and interpret features of data (Miles & Huberman 1994). Secondary coding or axial coding was achieved as the team put together preliminary codes and looked further into the data to see if there were any connections or additional categories that could have emerged from the codes. Selective coding was done by looking beyond what could have been obvious or missed during open and axial coding. Strauss and Corbin (1990) argue that the selective code is achievable if researchers avoid being married to the original codes and themes that emerge and open their eyes to the nuances that were less obvious and unanticipated. Subsequently, the research team gathered literature which enhanced discussions around the themes.

### Ethical consideration

Approval for this project was obtained from the Human Sciences Research Council (HSRC)'s Research Ethics Committee and the Associate Director of Science of the National Centre for HIV/AIDS, Viral Hepatitis, STD and TB Prevention at the USA's Centers for Disease Control and Prevention (CDC) in Atlanta, USA. An Information Sheet was made available to all participants. In addition, an Informed Consent Form was made available for youths and adults as well as parents/guardians of children aged 12–17 years, while an Informed Assent Form was made available for children aged 12–17 years.

## Findings

### General perceptions about HIV testing in different communities

Overall, the study found that there was some consensus among Africans, Indians and Coloureds who were mostly concerned about the low prevalence of HIV testing; however, Whites mostly felt that HIV testing was common in their community. The African participants across all age groups felt that there was very little self-initiated HIV testing in their communities. ‘Very few people test for HIV in my community’ reported a female adolescent from the Cape Town FG. Another female African adolescent from the same FG quantified the testing rates in her community as ‘Only 30% have tested around here, I would say.’ In an FG of adult African participants in Cape Town, similar perceptions about low HIV testing were indicated by a male adult participant who said that ‘Not even, probably less than 5%’*.* When the African youth were asked whether people in their community were testing for HIV voluntarily, they unanimously indicated that it was not the case at all. When asked if they themselves had voluntarily gone for an HIV test, they also indicated that they had not done so.

The Indian youth FG in Durban also reported that there was little self-initiated HIV testing in their community. A participant in this FG said that ‘Personally, I think it's a small proportion, I think, that actually go out for testing.’ Another female participant in the same FG said that ‘I think, because maybe they are not so exposed to it [HIV].’

There was a lot of scepticism among participants in the Coloured adolescents FG and when asked the same question, similar sentiments to those of participants in the Indian youth FG were expressed. One of the participants in one Coloured FG even emphatically stated that ‘I won't do it, I won't do it.’

Participants in the White FGs reported that many members of their community have tested during school campaigns and most people in their communities donate blood regularly. ‘A lot of people think that if they are not sexually active, then no risk, so they won't be tested,’ said one participant from this group. Similar views about not testing because of perceived low risk to HIV infection were shared by the White youth and adults groups. ‘It is not common with us here, I think very few people maybe have it [HIV],’ said an adult female in the White FG.

### Factors influencing testing in different communities

Table 1 presents themes that influenced participants’ views towards HIV testing that emerged from the study among different racial groups in Cape Town and Durban.

**Table 1.** Themes that have positive and negative influences towards HIV testing that emerged from different communities in Cape Town and Durban.

Themes	Encouraged HIV testing
Testing and blood donation campaigns	• Promote awareness
	• Dispel fear
	• Promote testing
HIV knowledge and awareness	• Understanding HIV and its transmission
	• Understanding prevention
Perceived benefits of testing	• Promote better quality of life
	• Ability to plan for the future
	• Freedom from worries about HIV
	• Access to treatment
	• Prevent the spread of HIV
Confidentiality	• Encourages testing
	• Protects from stigmatization and discrimination
	• Prevent victimization
	Discouraged HIV testing
Risk perception	• HIV risk attributed to other racial groups
Fear of testing positive	• Positive diagnosis
	• Becoming ill
	• Dying of AIDS
Stigmatization and discrimination	• Negative reaction from family, friends and community if positive
	• Isolation

#### Testing campaigns and blood donations

Overall, most White FG participants reported that members of their community regularly donated blood and participated in testing campaigns. The act of donating blood was seen as an expression of confidence that the blood is not infected with HIV. These perceptions of HIV testing were common among the White groups in Durban and Cape Town and across the age groups. The other FGs felt that voluntary HIV testing in their communities was a dreaded activity and HIV testing was associated with a positive HIV status. The concept of donating blood did not emerge in the discussions of the African and Coloured participants. Among the adolescent FGs with participants from both the White and Indian communities, the point was reiterated that in schools and college campuses calls from blood donation campaigns were the major drivers that encouraged people to take up voluntary HCT. For example, a female participant from the adult White FG said ‘So especially, because when we were students … the HIV Testing message was pushed so heavily and like, you were terrified not to have an HIV test’. Another female participant from the White adolescent FG commented that ‘everyone that donates blood gets tested and so I think, most, the majority of our school have been tested, about 80%’, and another female adolescent in this same group supported this opinion by adding ‘80% and that's because of the blood drive’. Another female participant in the White adolescent FG highlighted the issue of blood donation by saying: ‘And everyone that donates blood gets tested and so I think, most, the majority of our schools have been tested, about 80%. So like, it gets encouraged a lot to know your status and everything in our area.’

The Indian youth FG also mentioned that there were campaigns on their campuses that encouraged students to go for HIV testing, but there was no acknowledgement that they themselves had partaken in these campaigns. They only pointed out that a lot of people participate in these campaigns. One male participant from the same group said that:

I know that amongst the students that I talk to and friends that are sexually active, many, they know about AIDS, they know about condoms, but they think they're using the adequate techniques or mechanisms to avoid risk like using the condoms and participate in the blood campaigns.

Among the White adult FG it was indicated that among employed people from their community, testing was mostly done as a job requirement and for insurance policy purposes. ‘I think the other thing as well is when you have to take out life policies; you have to go for HIV testing, so there will be a fair number of people that would have had HIV testing’ said a male participant from the White adult FG.

#### HIV knowledge and awareness

A number of participants from the African youth FG felt that having in-depth knowledge would encourage them to test for HIV. ‘I think that maybe a person who is HIV positive and living with the virus for years can tell people and to also show them a person who is positive can lead a happy life,’ said one female participant from the group. A lack of clarity on how the HIV testing process happens can be seen as contributing to how people cognitively process the act of testing. A young female participant from the same group said that ‘If you are not told your status is positive or negative and then you find out at a later stage that you are positive by then the virus would have caused a lot of damage.’

A male participant from the White youth FG also shared similar sentiments in agreement with his peers:

If you're positive and you don't know, then what happens if you do have like sex with somebody else and then you, and you get them positive as well. Then you've got to live with that guilt … You know, I'd rather know so then that wouldn't happen.

Similarly one female from the Coloured adolescent FG also felt that it was important to know one's status: ‘You know, if you are a sexually active person, get to know your status, and do not be scared. You know what you are doing, so if you are using protection or not, take the test.’

#### Perceived benefits of testing

Participants in most FGs made reference to the fact that testing and knowing one's status come with the benefits of treatment and changing one's lifestyle. A male participant from the White youth FG stated that ‘testing and knowing ones status means that there is a lifestyle change that needs to happen, basically know your status and you can do something like taking ARVs [antiretrovirals]’*.* Another male participant from the same FG also asserted that ‘surely, the consequences of not knowing, are far worse than knowing … basically know your status and you can do something like taking ARVs’.

A similar view to that indicated previously was expressed by participants from the African youth FG. For example, one female participant from this FG felt that testing was beneficial for her peers. She stated that: ‘Now she will know whether she has the virus or not. Then she will know how to conduct herself in a proper manner and take medication.’ The same view was expressed by participants in the Coloured adolescent FG. One female participant from this group shared the same sentiments by saying the following: ‘Also if I was positive I'd want to start living a life of, I'd like want to start living that, a life of like, of like no one … like correcting things that was like wrong.’ Similarly a male participant in the same group agreed by saying that ‘I would just like want to start living a healthier life, like for myself and like for people around me.’

#### Confidentiality and privacy

There was general agreement that confidentiality and privacy were positive attributes for HIV testing among all 12 FGs. A male participant from the White youth FG felt strongly about privacy and not letting people know the purpose of your consultation with health workers by saying that: ‘You can go in there for whatever reason, to get medicine or to get tested. So nobody knows if you've been tested, or not, except you and the person testing you.’ A female participant from the African Youth FG also agreed with these sentiments by stating that ‘I say that a person must be tested alone … I think a person should hide and do it alone’*.* A similar view was expressed by a participant from the Coloured adolescent FG as follows: ‘I think that the young people would prefer to be alone when they do the testing. It's a matter of privacy; people will want to keep all information private … confidential.’ A male participant from the Indian adolescent FG also reinforced that ‘at this age we want everything to be private like HIV status’.

### Factors hindering testing in communities

#### Inaccurate risk perception

The perception of not being at risk of HIV as a reason for not testing was also expressed by participants in most White and Indian FGs. A female participant from the Indian youth FG said that ‘they are not educated or assume that maybe it's not them who would have the virus, so to speak’. A similar view was expressed by a female participant in the Indian adult FG who stated that:

Some people are just ignorant to it and you know, even though they are sexually active and maybe they are not using any protection, but they still, you know, wouldn't go, would refuse to go for these tests, because they think that I would not get infected and I think there's also this portrayal that because it's a so-called African disease and it's mainly Black people that are affected with it.

Two White female adults felt that the issue of HIV testing was for other racial groups, and their group interaction went as follows: ‘ … in my matric class 4 Coloured girls fell pregnant and no White girls were pregnant so I think it's also the fact that maybe the White middle class, they use protection … ’.

#### Fear of testing HIV positive

There was a strong fear of the outcome of testing among the African and Indian FGs and this was reinforced by a belief that once tested it was inevitable that one would be found to be HIV positive. A female African participant in the youth FG conveyed this fear of being and knowing that one is HIV positive by saying ‘They are afraid of getting their results because when a person is told that he is HIV positive he just gets afraid to accept that and so they prefer not to know anything at all.’ This was a response to a question of what makes people not voluntarily go for HIV testing. On the other hand, the participants in the White FGs felt that they were more informed about safe sex practices and this fear of HIV testing was not common. The following excerpt from the transcripts is part of the FG conversation between the researcher and the white adolescents (12–17 years) from a mostly White populated school:

Researcher: And you said the opposite and you are, it is like 5%. So why do you think it is so low in your area?

Male adolescent: I think in the school area that I am in, children and teenagers are, have the knowledge about HIV and AIDS and know that, being sexually active, that you can be (infected) by it and I think they are just more knowledgeable as in your school it might be more, you know, they might be more sexually active. (Laughter)

This extract demonstrates that there is a belief that the White community is more informed about the sexual risks of spreading HIV, are taking precautions and therefore not afraid of taking an HIV test and getting the results.

Some participants in the African youth FG highlighted that people in their community, particularly men, were afraid of the HIV test. For example, one female participant in this age group said that ‘They are afraid of getting their results because when a person is told that he is HIV positive he just gets afraid to accept that and so they prefer not to know anything at all.’ Another participant in the same group highlighted that men in particular were reluctant to test and said

Because they do not want to be revealed since they believe that this disease came with women because it is said that when you are pregnant you must go and check, they get surprised when a woman comes to him and says I went to check and they said I am HIV positive, I think you should also go and check, he does not want he would rather run away, also when they are afraid they run away and end up not testing in the end.

Another male participant from the Coloured adolescent FG also said that ‘It isn't just to go and test, you feel everything, you never know – because they say a piece of paper can upset your life.’

Apart from fear, HIV testing was also at times associated with stress, especially among participants in FGs from African, Coloured and Indian communities. One female participant from the African youth FG indicated that the reason for people not testing is that ‘They are afraid of the stress, they are afraid it will affect their work.’ A male participant from the Indian youth FG said that ‘Well, the basic thing is fear, because people are afraid to die. So if they find out, it's like a guarantee that they are going to die. Their life is going to be shorter.’ A male participant from the Coloured adolescent FG reiterated this view by saying that ‘Some of them get scared. They think “death.” Death is coming.’

#### Stigma and discrimination

A major issue of concern expressed by FG participants in this study was the ill treatment by family, friends and community when they learn that one is HIV positive. The fear of stigma and discrimination seemed to be strongest among the Indian community across all age groups. For example, a male participant in the Indian adult FG said that

I think also the fear would be that if people did find out … you would be ostracized to a certain extent because of people's fear, even though everyone knows like you can't get it from like touching people whatever. Like people are going to treat you differently and are going to possibly touch you less, be like think twice before hugging you and things … people are not going to let you look after their kids or you know things like that.

A similar view was also expressed among the African FGs. For example, a female participant in the African youth FG said that

A person may be afraid to say that they are HIV positive because they will start to separate the dishes, they will pull the children closer or when he/she calls a child to play they will just say to the child don't go there he/she is positive.

Finally, going for an HIV test by Indian adolescents was an acknowledgement that one is sexually active and this, according to their group discussion, was something that is frowned upon by their parents and community. One female participant in the group said that

Ja I think It is more a thing where if you are going for an HIV test its going to be stigmatized that you are sexually active and a lot of us youngsters do not want people to think that and know that.

## Discussion

In spite of the majority of South Africans, especially women, having reported to have had ever been tested for HIV in 2012 (see Shisana *et al*. 2014) and the resounding success of the recent national HCT campaign as reported recently by Mbengashe *et al*. (2012), our study found that there was general consensus among African, Coloured and Indian FGs in Durban and Cape Town that the number of people testing for HIV in their respective communities was relatively small. This perception clearly contradicts the findings reported by Shisana *et al*. (2009, 2014), which showed that Africans had the highest rate of HIV testing compared to other population groups. For example, 73.3% of the 28,997 people who volunteered to have their blood specimen taken in the 2012 national HIV survey were African (Shisana *et al*. 2014). In the present study the participants in the African FGs expressed the least willingness to give blood and to go for HIV testing than did their Coloured, Indian and White counterparts in other FGs. Thus, the perception that Africans are not taking up HIV testing calls is not consistent with the quantitative reports of the HCT campaigns and the national HIV surveys as have been indicated. The White FG participants, on the other hand, expressed a greater willingness to donate blood than to take an HIV test; this reflects low testing rates among the White population as seen in previous national HIV surveys (also see Shisana *et al*. 2005, 2014; Shisana & Simbayi 2002). Our results suggest that perceptions about HIV testing in the various communities except Whites were still largely negative.

This perceived lack of willingness to test for HIV might be associated with what Malcom, Aggleton, Bronfman, Galvao, Mane and Verrall (1998) describe as the fear of being stigmatized and discriminated. While it is acknowledged that prevention is the key strategy to overcoming the wide spread of HIV, the epidemic fear of being stigmatized severely constrains the individuals, families and communities who want to access services (Malcom *et al*. 1998:4). Kalichman and Simbayi (2004) also state that stigmatizing beliefs about HIV/AIDS and associated fear of discrimination often influence the decision to seek HIV testing and other related services.

We found that the reasons that discouraged or encouraged HIV testing varied from one community to another (see summary in Table 1). An important factor that encouraged HIV testing included blood donation campaigns, especially among the White communities. There was also an indication that knowledge about HIV transmission and prevention encouraged individuals to test and take their results. Seemingly, assurance of confidentiality around HIV testing was a critical factor in encouraging people to test. Confidentiality is one of the principles recommended by the WHO for HCT (WHO 2011). The emphasis of confidentiality throughout the HIV testing process encourages respondents to agree to test for HIV and to feel comfortable with the person doing the test.

Our analysis suggests that the decision to test or not test for HIV is not constructed only at the level of the individual but is constrained, influenced and facilitated by the social, cultural and environmental context in which individuals live their social and sexual lives. The different contextual factors inform behavioural and normative beliefs, social norms and peer social influence and perceived risk and vulnerability, all operating together to influence willingness to test for HIV. For example, with regard to the environmental context, the fact that African participants expressed a lack of willingness to test for HIV is not surprising, given that HIV prevalence is higher among black adolescents than among other race groups (Pettifor, Rees, Kleinschmidt, Steffenson, MacPhail & Hlongwa-Madikizela 2005; Shisana *et al*. 2009) and this could explain issues of fear, stigma and discrimination that discourage HIV testing (Kalichman and Simbayi 2004). Those who perceived themselves as less likely to be HIV positive felt less obliged to take the HIV test. Maharaj and Cleland (2011) also found that very few White participants expressed concern about HIV, and this was associated with high scores on abstinence and consistent condom use.

In the group discussions White participants suggested that they had more accurate knowledge about HIV and they also stressed the need for confidentiality when they needed to go for HIV testing. This is consistent with findings from Peltzer, Matseke, Mzolo and Majaja (2009) that Whites and Indians in the age group 25 to 34 years who had achieved an education of Grade 12 and above reported to be aware of their HIV status and had tested in the past year compared to their African counterparts. Our findings are also consistent with the findings from previous studies where it was found that both HIV/AIDS education and ensuring confidentiality consistently encouraged HIV testing (Solomon, van Rooyen, Griesel, Gray, Stein & Nott 2004). Interpretations of our findings should take into account the educational differences of participants as the African and Coloured participants were recruited outside of the school or university environment, while the White and Indian participants were recruited mostly from tertiary institutions. This education disparity can be a contributing factor to the different perceptions about HIV testing behaviour.

One of the interesting findings of this study is that there was a general perception among White and Indian communities that they are safe from HIV and that it was not much of a problem in their communities. Maharaj and Cleland (2011) also found that very few White participants expressed concern about HIV, and they associated this with high scores on abstinence and consistent condom use. There is also evidence that shows that both of these communities have the lowest HIV prevalence of less than 1% among people aged two years and older in the general population compared to their African and Coloured counterparts, where prevalence is at 15% and 3.1% respectively (see Shisana *et al*. 2014). Although low risk perception by Whites and Indians reflects the reality of the situation as evidenced by the very low HIV prevalence found in these communities, they should however not be complacent and have a false sense of security, particularly as HIV prevalence increased from 1.7% in 2008 to 3.1% in 2012 among the Coloured communities (see Shisana *et al*. 2014). Nduna and Mendes (2010) caution against ‘hiding behind the numbers’ for the population groups with low levels of HIV infections and also using this as an opportunity to fuel negative stereotypes about population groups struggling with fighting the HIV battle. Indeed there is evidence from previous research that low perceived risk of contracting HIV has hindered HIV testing in the context of HCT in Tanzania (Charles, Kweka, Mahande, Barongo, Shekalaghe, Nkya *et al*. 2009), while people with high self-perceived HIV risk were more likely to either be tested or express positive intentions regarding future testing (Lopez-Quintero, Shtarkshall & Neumark 2005). De Wit and Adam (2008) also stated that people are more likely to test if they had been involved in a behaviour they consider to be putting them at risk of HIV, or if they felt that HIV testing had a potential benefit of starting treatment.

Our study found that the fear of knowing one's HIV status and the fear of stigmatization were the most common reasons for not testing for HIV, particularly among African and Indian communities. The fear of knowing one's HIV status has been shown to originate from perceiving an HIV-positive result as a death sentence and from the anticipation of severe stigma (De Wit & Adam 2008; Jurgensen, Tuba, Fleykesnes & Blystad 2012). HIV-related stigma and discrimination have been identified as one of the major barriers to HIV testing throughout sub-Saharan Africa (Ogden & Nyblade 2005). Stigma has been a component of the HIV/AIDS scenario since the onset of the epidemic and has a significant and harmful effect on health and disease transmission by delaying individuals to seek the care that they desperately need (Leclerc-Madlala *et al*. 2009). Kalichman and Simbayi (2003) also found that it was mainly people who have never tested for HIV that had reservations about HIV testing and that ascribed greater shame, guilt and social disapproval of PLWHA. Similar findings were reported by Genberg, Hlakuva, Konda, Maman, Chariyalerstak, Chingono, *et al*. (2009) who found that, in a study conducted in South Africa and three other countries, HIV-related stigma acted as a barrier to HCT as well as to accessing effective prevention and care services. In addition, studies among PLWHA in South Africa have revealed fear related to stigma (Abrahams & Jewkes 2012), especially among women (see Leclerc-Madlala *et al*. 2009). Various studies have also shown that internal stigma among PLWHA can be stronger than external stigma, that is, stigma from other people (see Cloete, Kalichman & Simbayi 2013; Cloete, Simbayi Kalichman, Strebel & Henda 2008; Simbayi, Kalichman, Strebel, Cloete, Henda & Mqeketo 2007).

Finally, the finding that men shun HIV testing is not surprising. Similar findings have been reported by various studies conducted in a number of African countries including South Africa (e.g. see Coates, Kulich, Celentano, Zelaya, Chariyalertsak, Chingono, *et al*. 2014; Shisana & Simbayi 2002; Shisana *et al*. 2005, 2009, 2014; Van Rooyen, McGrath, Chirowodza, Joseph, Fiamma, Gray, *et al*. 2013; Wilson, Strebel, Simbayi, Andipatin, Potgieter, Msomi, *et al*. 2001) and Kenya (e.g. see National AIDS and STI Control Programme, Ministry of Health, Kenya 2013). This is because men's health-seeking behaviour is different from that of women. Indeed, there is cross-cultural evidence available which suggests that men often delay health care seeking as a result of gender norms and ideas of hegemonic masculinity, where toughness and self-reliance are valued (Courtenay 2000; O'Brien, Hunt & Hart 2005). Consequently, men often put off health care seeking, especially at public health care centres, because they adhere to hegemonic masculinity ideals of ‘toughness’, while women seek help much earlier as it a normalized behaviour, especially as women are accustomed to health care seeking for reproductive purposes. This has led to unequal access to ART with about twice as many females accessing treatment than their male counterparts in South Africa during 2012 (see Shisana *et al*. 2014). Furthermore, in the area of HIV this is further exacerbated by the fact that women often attend antenatal and PMTCT programmes and get tested before men. Consequently, women get blamed unfairly of infecting their husbands or male sexual partners because they find out about their HIV status first (see Petros *et al*. 2006). Indeed, Project Accept (HPTN 043), which implemented a multi-component, multi-level social and behavioural prevention strategy including using mobile clinics, improved the uptake of HIV testing among men by as much as 45% (Coates *et al*. 2014; Van Rooyen *et al*. 2013). This suggests that health care seeking behaviour and especially HIV testing might be improved, especially among men, if services are made available in non-clinical settings such as mobile clinics and work places (Shisana *et al*. 2014; Van Rooyen *et al*. 2013).

Some limitations of this study were noted; firstly, the snowball recruitment approach that we used can include an element of bias in that the researchers and the participants recruit only people who are familiar to them or within their networks. These people might share common views and have similar perceptions about social issues. Secondly, although qualitative methods allow for a more in-depth understanding of issues under investigation, qualitative methods in general and FGs in particular do not allow for generalizing the findings obtained to larger populations as the participants are not meant to be a representative sample.

## Conclusion

This study provides an indication of the perceptions about the acceptability and prevalence of HIV testing and the factors that influence them in the four different racial communities found in South Africa. The intention of this study was not to paint a picture of disadvantaged or advantaged communities, but rather to explore and establish behavioural nuances that might be pertinent to specific sociocultural settings. These group responses have indeed shown us that South Africa is not a homogenous society in terms of their thinking about HIV testing, and therefore care and nuance should be applied when designing interventions. Equitable but content relevant approaches can only be designed if information such as that found in this study is acquired and taken into consideration.

The findings from the present study shed light on the complex factors that influence HCT uptake in the various racial groups in South Africa. In general, inaccurate risk perception, perceived lack of confidentiality and privacy, fear and anxiety about having a positive HIV test and HIV-related stigma and discrimination all affect perceptions about HIV testing, especially among Africans and Coloured communities. Culturally influenced sexual norms among Indians remain major barriers to HIV testing, while HIV knowledge and awareness, being a blood donor and perceived benefits for testing encourage HCT especially among Whites and to some extent among Indians. Lower risk perception was also found among Whites and Indians. All the results reiterate the importance of spreading messages that encourage HIV testing and ensuring confidentiality for HIV testing in communities, especially where there is stigma attached to PLWHA.
